# Leiomyosarcomas of the inferior vena cava: diagnostic features on contrast-enhanced CT, ultrasonography and MRI

**DOI:** 10.3389/fonc.2025.1442674

**Published:** 2025-02-04

**Authors:** Xiaolin Xu, Shilei Zhao, Lifang Xue

**Affiliations:** ^1^ Department of Ultrasound, Beijing GoBroad Hospital, Beijing, China; ^2^ Department of Radiology, Peking University International Hospital, Beijing, China

**Keywords:** CT, ultrasonography, MRI, leiomyosarcomas, inferior vena cava

## Abstract

**Purpose:**

To evaluate clinical presentation and imaging characteristics of leiomyosarcomas of the inferior vena cava (IVC LMS) using contrast-enhanced CT (CECT), ultrasonography (US), magnetic resonance imaging (MRI), and to identify features that facilitate early and accurate pre-operative diagnosis.

**Materials and methods:**

Our study enrolled 21 patients with pathologically confirmed IVC LMS from October 2015 to June 2022. All participants underwent CECT, and additionally, 12 participants had US examinations and 3 had MRI. Images were independently reviewed by two experienced radiologists. The clinical presentations and diagnostic characteristics were recorded.

**Results:**

The study involved 16 female and 5 male patients, with an average age of 55 ± 11 years (ranging from 34 to 80 years). Common clinical symptoms included abdominal pain, back pain, leg discomfort, abdominal distension, jaundice, and the presence of an abdominal mass. On CT scans, a large, lobulated, heterogeneous mass with progressive enhancement was typically seen in 13 of the 21 patients (61.9%). Ultrasonography revealed that IVC LMS typically presented as a lobulated, heterogeneous, hypoechoic mass. Color Doppler imaging evaluated lumen obstruction in 8 of the 12 patients (66.7%), and high velocity flow signals were detected by Pulsed wave Doppler in 4 of the 12 patients (33.3%). On MRI, IVC LMS presented as a heterogeneous mass that exhibited intermediate intensity on T1-weighted images, slightly high intensity on T2-weighted images and high intensity on diffusion-weighted images.

**Conclusion:**

Several diagnostic characteristics on CECT, US and MRI could aid in the diagnosis of IVC LMS. The detection of a heterogeneous mass with progressive enhancement along the inferior vena cava on CECT was strongly indicative of IVC LMS. Both CT and US are effective in accurately indicating the location of the tumor within the IVC.

## 1 Introduction

Leiomyosarcoma of the inferior vena cava (IVC LMS) is a rare vascular sarcoma that originates in the smooth muscle layer of the media (1). Despite its rarity, it is the most frequently occurring malignant tumor in the IVC. The first case of IVC LMS was documented in 1871 (2). Often, patients are asymptomatic or display nonspecific symptoms in the early stages, leading to a diagnosis usually at an advanced stage. The prognosis is generally considered poor due to the high likelihood of local recurrence and metastasis (3). Early detection is desirable and may be achievable with effective radiological techniques. Contrast-enhanced CT (CECT) and magnetic resonance imaging (MRI)are more accurate than ultrasonography (US) in determining the full extent of IVC LMS, pinpointing the tumor’s origin, and assessing its proximity to local structures, such as the gastrointestinal tract, which benefits from CT’s insensitivity to interference by intestinal gas (4). CECT is predominantly used for diagnosing the disease and for monitoring after surgery. MRI is more accurate than CECT in identifying the origin location due to its superior soft tissue resolution. US, while being cost-effective, free of radiation, and repeatable for measuring tumor size, can also display vessel distension and tumor vascularity. However, it is less reliable for distinguishing benign from malignant tumors (5). Currently, there are only limited studies that detail the radiological imaging characteristics of IVC LMS, mostly derived from case reports. There have been some reports on CT and a few on MR imaging of IVC LMS but publications on US are relatively rare (5–10). The aim of this study was to highlight the imaging characteristics of IVC LMS, identifying key findings that could aid in early diagnosis and discussing differential diagnoses.

## 2 Materials and methods

### 2.1 Patients

Our institutional review board approved this retrospective cohort study, and informed consent was waived. The study included 21 consecutive patients from 15 October 2015 to 13 June 2022, all of whom had histologically confirmed leiomyosarcoma of the inferior vena cava (IVC LMS) through surgery or biopsy. We also recorded the ages, sex, and initial symptoms of the patients.

### 2.2 US technique

US examinations were conducted using a Philips iU 22 (Philips Medical Systems) machine. We recorded various ultrasonic characteristics of the tumors, including echogenicity, contour, presence of necrosis, flow velocity within the IVC, and whether there was any local invasion or obstruction of the IVC lumen.

### 2.3 CT technique

CT was performed on either a dual-source CT scanner (Siemens Definition Flash; Siemens Healthcare) or a 128-row CT scanner (Siemens Definition AS; Siemens Healthcare). The scanning protocol included a section width of 1 mm, a reconstruction interval of 1 mm, and a pitch of 1. Both abdominal and pelvic CT scans encompassed plain scans as well as arterial and venous phase imaging, conducted before and after the intravenous administration of the contrast agent iohexol (Yangzijiang Pharmaceutical Group) at a flow rate of 3 ml/s and a dosage ranging from 80 to 100 ml.

### 2.4 MRI technique

MRI examinations were performed using a 3.0-T system (GE SIGNA Pioneer, GE Medical Systems, Milwaukee, WI, USA), and included unenhanced imaging consisting of in-phase and opposed-phase T1-weighted images, fat suppression T1-weighted images, T2-weighted images, high-resolution T2-weighted black blood sequences with and without fat suppression, and diffusion-weighted images.

### 2.5 Review of the medical records and images

The initial CECT and US images were independently evaluated by two experienced radiologists, who had 34 and 7 years of experience in abdominal imaging diagnosis, respectively. A consensus opinion was achieved between them. The IVC was categorized into three segments for analysis: Segment I, located below the renal veins; Segment II, extending from the hepatic veins to the renal veins; and Segment III, running from the right atrium to the hepatic veins (10). For the CECT images, recorded findings included the lesion’s location, degree of enhancement, maximum diameter (size of the tumor), presence or absence of local invasion, and the presence and location of distant metastases. The CT attenuation values were quantitatively measured across the plain scan, arterial phase, and venous phase. Tumor attenuation was assessed by placing an oval region of interest, measuring 10 mm^2^, within the tumor on each phase of the image sets, while avoiding calcifications, necrotic areas, and blood vessels. In cases of intratumoral heterogeneity, measurements focused on the area representing the predominant (>50%) tumor enhancement pattern. Lesion enhancement patterns were classified into three types: progressive, persistent, and wash-in and wash-out. The progressive enhancement pattern was identified when the degree of enhancement increased by more than 10 HU from the previous phase. The persistent enhancement pattern was noted when the enhancement in the arterial phase was followed by less than a 10 HU increase in the venous phase. The wash-in and wash-out pattern was recognized when enhancement in the arterial phase decreased by more than 10 HU in the venous phase.

## 3 Results

### 3.1 Clinical features of the study cohort

Among the 21 patients studied, 16 were female and 5 were male. The average age of the cohort was 55 years, with a range from 34 to 80 years. The mean age for female patients was 53 years (ranging from 34 to 68 years), while male patients had a mean age of 60 years (ranging from 39 to 80 years). The mean value of body mass index was 22.1, with a range from 14.3-29.1. Three patients (14.3%) had a history of alcohol consumption and smoking. No patients had diabetes or coronary heart disease. The summary of the clinical symptoms is presented in **Table 1**. Out of the 21 patients, 20 exhibited symptoms: 18 experienced abdominal pain, four reported back pain, three had leg discomfort, one presented with both abdominal distension and jaundice, and another had an abdominal mass.

**Table 1:** Overview of clinical symptoms in 21 IVC LMS patients.

Clinical Symptoms	Number	Case Number
Asymptomatic Abdominal pain Back pain Leg discomfort Abdominal distension Jaundice Abdominal mass	1 18 4 3 1 1 1	18 1,2,3,4,5,6,7,10,11,12,13,14,15,16,17,19,20,21 2,10,16,21 1,8,11 9 9 1

### 3.2 CT features

**Figure 1:**
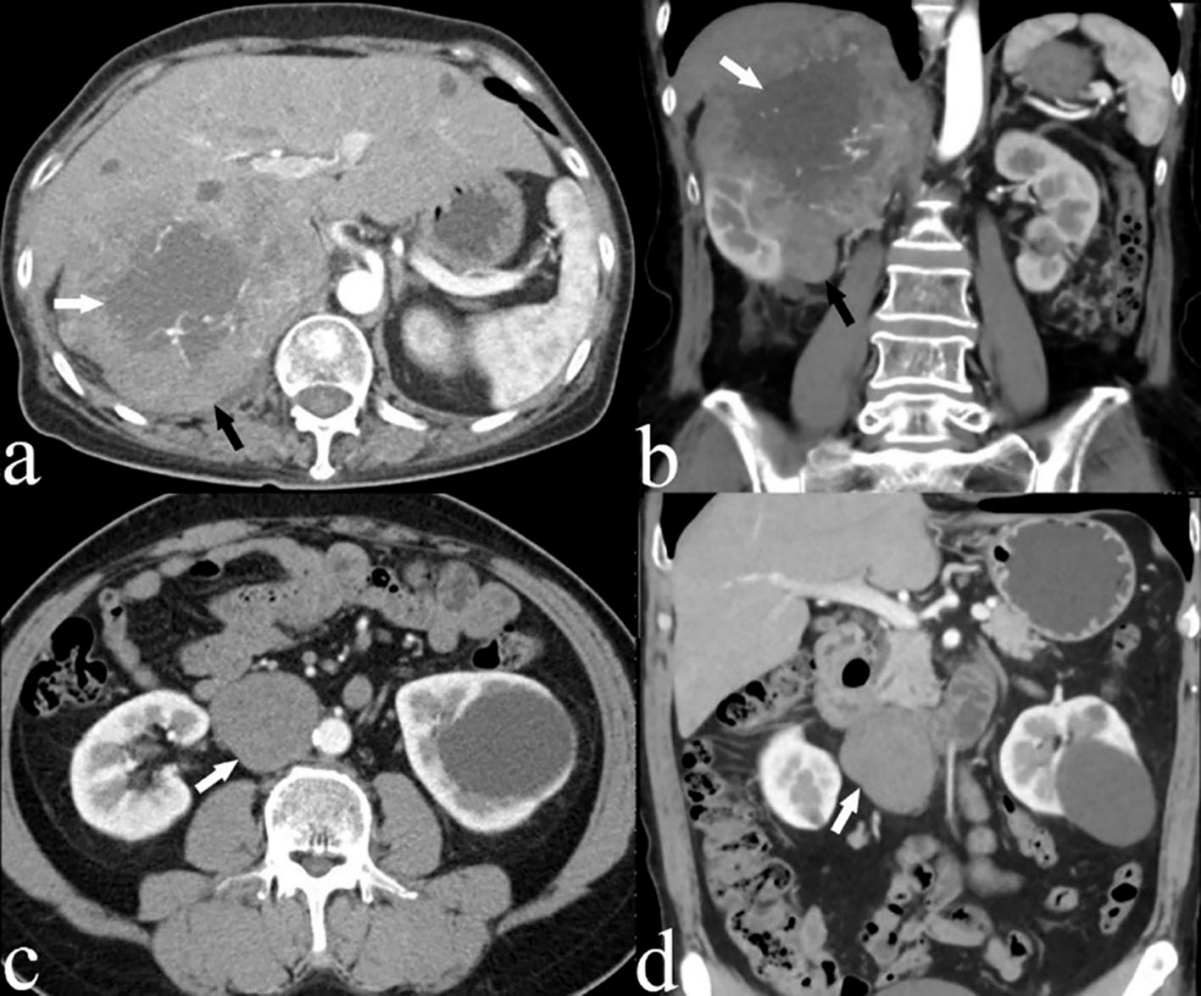
**(A)** Axial and **(B)** coronal CECT images of a 66-year-old woman (case 3) depicting a large, heterogeneous leiomyosarcoma in segment II of the IVC (black arrow), with an imperceptible IVC lumen. Areas of low attenuation suggest necrosis (white arrow). **(C)** Axial and **(D)** coronal CECT scans of a 54-year-old male patient (case 4) displaying an oval, homogeneous leiomyosarcoma in the IVC (white arrow).

**Figure 2:**
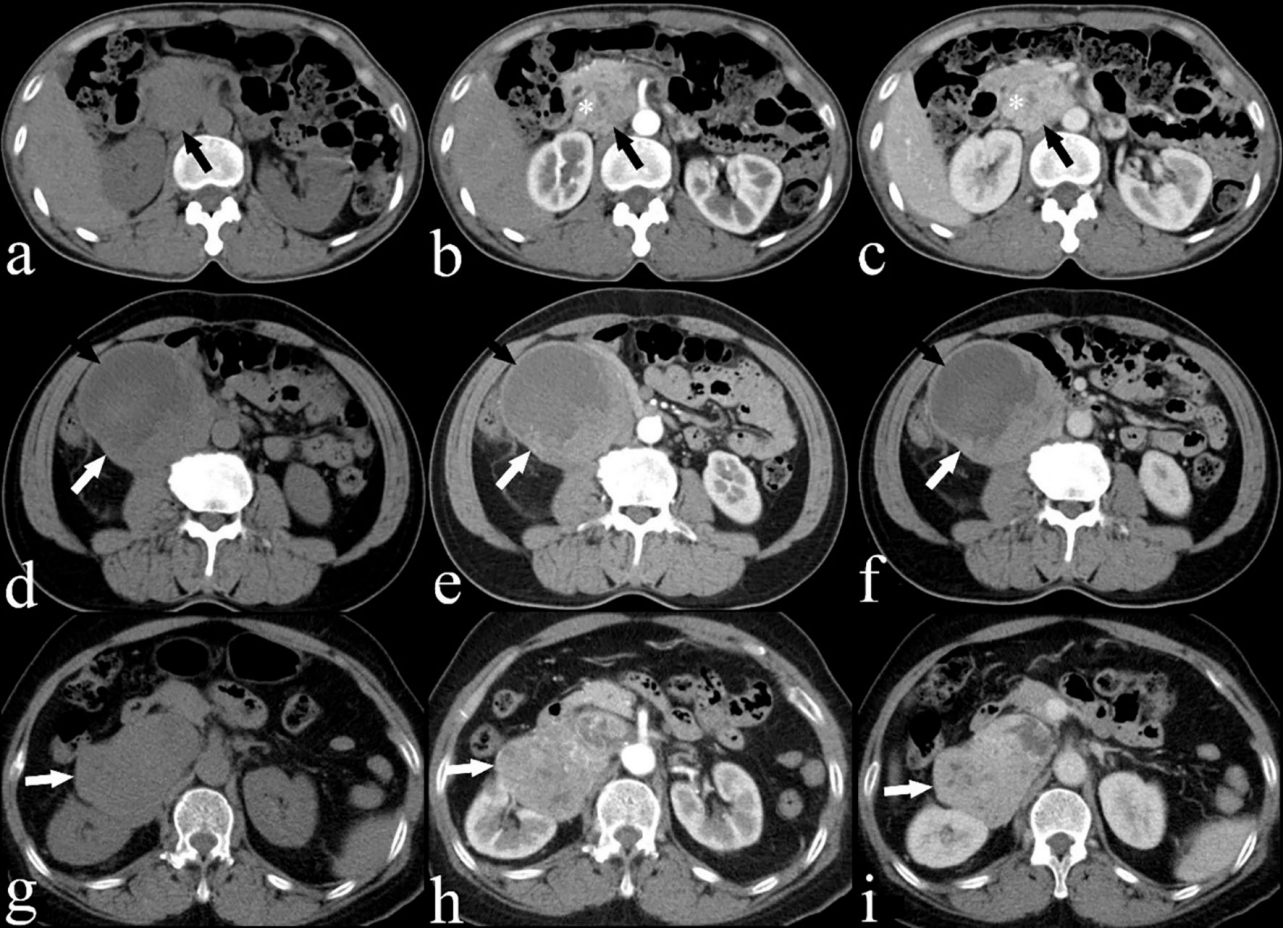
**(A)** Plain, **(B)** arterial and **(C)** venous CECT images of a 53-year-old woman (case 10) showing a heterogeneous IVC LMS (black arrow) with a progressive enhancement pattern and extension into the IVC (*). **(D)** Plain, **(E)** arterial and **(F)** venous CECT images of a 65-year-old man (case 7) showing a heterogeneous IVC LMS (white arrow) with a persistent enhancement pattern, including areas of low attenuation suggesting necrosis (black arrow). **(G)** Plain, **(H)** arterial and **(I)** venous CECT images of a 67-year-old woman (case 15) showing a heterogeneous IVC LMS (white arrow) with a wash-in and wash-out enhancement pattern.

**Figure 3:**
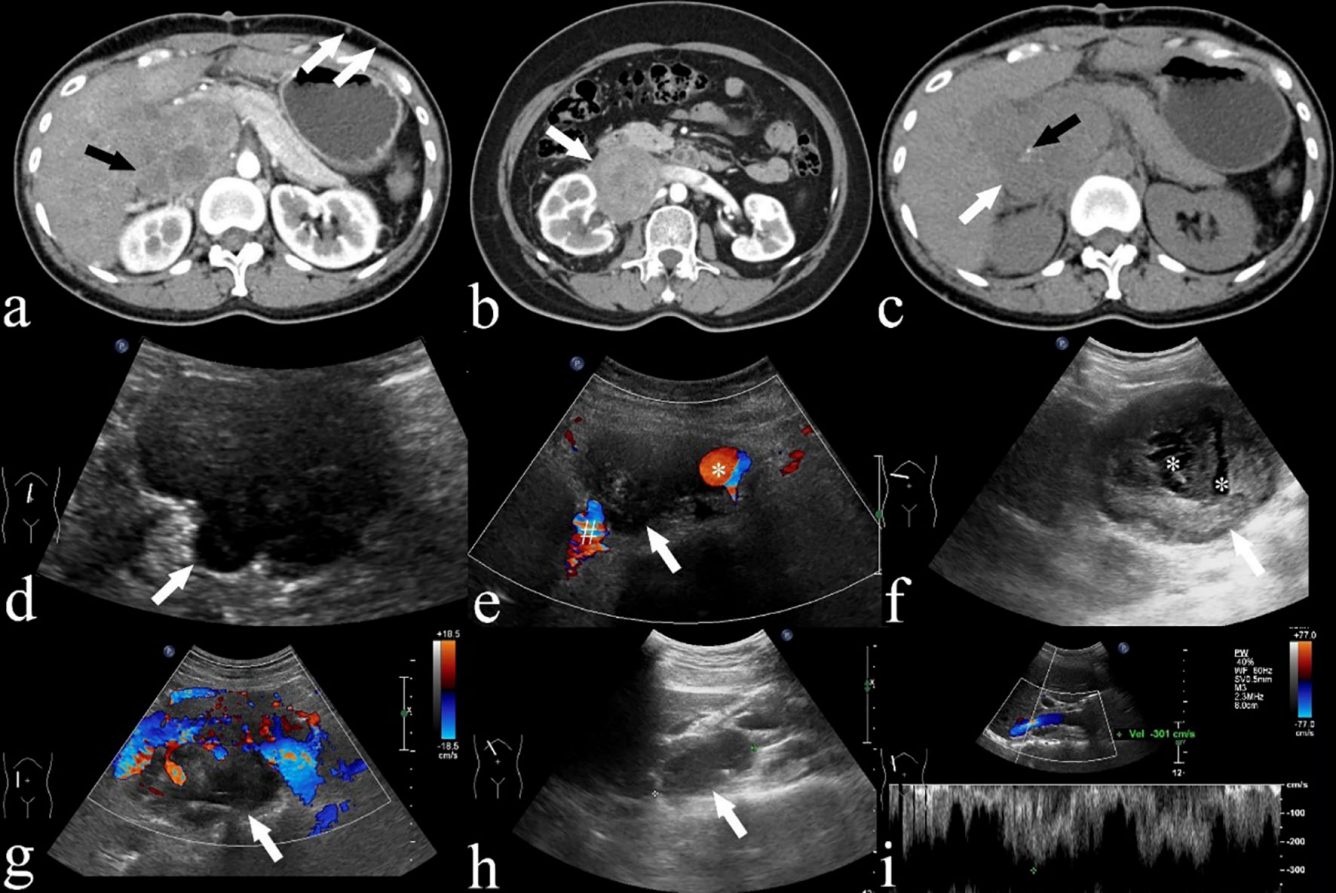
**(A)** A CECT of a 42-year-old woman (case 18) demonstrating an IVC LMS in segment II of the IVC (black arrow), distending the IVC. Despite being asymptomatic, the patient presents with extensive collateral formations (white arrows). **(B)** A CECT image of a 53-year-old woman (case 12) displaying a low attenuation mass (white arrow) extending into the right renal vessel and involving segments I and II. **(C)** A CECT image of a 42-year-old woman (case 18) demonstrating a low attenuation mass (white arrow) with punctate calcification (black arrow). **(D, E)** Ultrasound images of a 68-year-old woman (case 6) depicting a lobulated hypoechoic mass (white arrow) with extension into IVC (#) and aorta (*). **(F)** Ultrasound image of a 68-year-old woman (case 7) exhibiting an oval hypoechoic mass (white arrow) with anechoic areas suggesting necrosis (*). **(G)** Ultrasound image of an 80-year-old man (case 11) displaying an oval hypoechoic mass (white arrow) extending into IVC. **(H, I)** Pulsed wave Doppler images from a 34-year-old female (case 20) reveal high velocity flow in the IVC due to tumor compression.

All 21 patients in the study underwent CECT, and the findings are summarized in **Table 2**. CECT images revealed that 20 of the 21 patients (95%) had an oval, lobulated, irregular, and heterogeneous mass (**Figures 1A, B**), while only one patient (5%) displayed a homogeneous mass (**Figures 1C, D**). The average size of the tumors was 8.5 cm, with a range from 4.3 to 16.7 cm. The attenuation values for IVC LMS ranged between 35 HU and 49 HU, with a mean value of 41.5 HU. In terms of enhancement patterns, CECT showed that the tumor parenchyma exhibited a progressive enhancement pattern in 13 patients (61.9%) (**Figures 2A–C**), a persistent enhancement pattern in six patients (28.6%) (**Figures 2D–F**), and a wash-in and wash-out pattern in two patients (9.5%) (**Figures 2G–I**). The attenuation values in the arterial and venous phases ranged from 52 HU to 123 HU and 50 HU to 135 HU, respectively, with mean values of 71.2 HU and 89.3 HU. CECT also indicated tumor extension into adjacent organs: 12 into the renal vessel, nine into the duodenum, six into the aorta, four each into the kidney and liver, and other extensions including two each into the iliac vessels, ureter, psoas major, pancreas, adrenal gland, and one each into the diaphragm, ascending colon, and ovarian vein. Extensive collateral formation and varices were observed in 16 patients (76%) (**Figure 3A**). The IVC lumen was obscured at the point of maximal tumor contact in 19 of the 21 cases (90.5%) (**Figure 1A**). Tumor locations were as follows: five in Segment II (**Figures 1A, B**), 12 in Segment I, and four involving both Segments I and II (**Figure 3B**). At presentation, three patients (9.5%) had metastases—two with liver metastases and one with metastases to the lung and adrenal gland. Necrotic areas were identified in 17 of the 21 cases (81%) (**Figures 1A, B**), and calcifications were present in two cases (**Figure 3C**).

**Table 2:** Summary of CECT findings of 21 IVC LMS cases.

Imaging Findings on CT	Number	Percentage (%)
Contour		
Irregular Smooth	6 15	28.6 71.4
Enhancement pattern		
Progressive Persistent Wash-in and wash-out	13 6 2	61.9 28.6 9.5
Invasion of local structures		
Present Absent	18 3	85.7 14.3
Metastases		
Present Liver Lung and adrenal gland Absent	2 1 18	9.5 4.8 85.7
Tumor location		
Segment I Segment II Segment I and II	12 5 4	57.1 23.8 19.1
Calcification		
Present Absent	2 19	9.5 90.5
Necrosis		
Present Absent	17 4	81 19

### 3.3 US features

US examinations were performed on 12 patients, with findings summarized in **Table 3**. All patients exhibited a hypoechoic mass on ultrasound. Of these, 10 of the 12 patients (83%) had a lobulated mass (**Figure 3D**), while 2 of the 12 displayed an oval mass (**Figure 3F**). Additionally, 8 of the 12 patients (67%) had a heterogeneous mass (**Figure 3D**). Central anechoic areas indicative of necrotic tumor tissue were present in 10 of the 12 tumors (83%) (**Figure 3F**). US also revealed tumor extension into adjacent organs and vessels, including involvement of the right kidney in 5 patients, the right adrenal gland in one patient, the IVC in 11 patients (**Figures 3E, G**), the renal artery and vein in 4 patients each, and the abdominal aorta in 4 patients (**Figure 3E**). Color Doppler imaging was utilized to illustrate the relationship between the tumor and adjacent vessels such as the IVC or aorta (**Figures 3E, G**). Stenosis of the IVC, suggesting lumen obstruction, was observed in 8 of the 12 patients (67%) using Color Doppler. Pulsed wave Doppler was able to detect high velocity flow signals, indicative of the tumor compressing the IVC in real-time (**Figures 3H, I**). High flow velocities ranging from 100 to 301 cm/s were recorded in 4 of the 12 patients (33.3%). The remaining 8 patients (67%) showed normal flow velocities ranging from 10 to 30 cm/s in the IVC.

**Table 3:** Summary of US findings of 12 IVC LMS cases.

Imaging Findings of US	Number	Percentage (%)
Echo		
Hypoechoic Homogeneous Heterogeneous	12 4 8	100 33.3 66.7
Contour		
Lobulate Oval	10 2	83.3 16.7
Central anechoic areas		
Present Absent	10 2	83.3 16.7
Invasion of local structures		
Present Absent	12 0	100 0
Lumen obstruction of IVC		
Present Absent	8 4	66.7 33.3
Flow velocity of IVC		
High flow velocity	4	33.3
Normal range	8	66.7

### 3.4 MRI features

**Figure 4:**
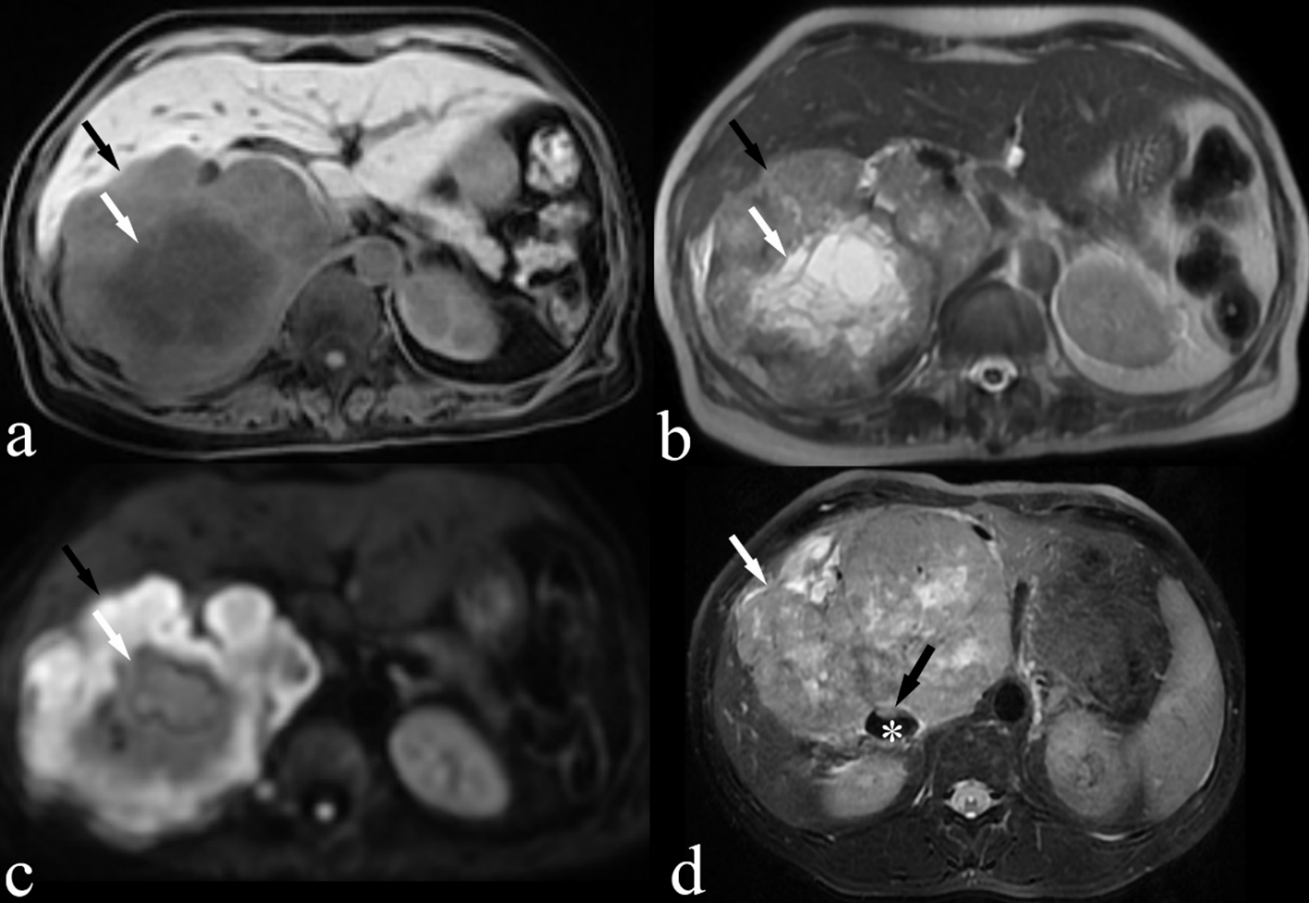
MRI images of a 66-year-old woman (case 3, panels **A–C**) and a 39-year-old male patient (case 9, panel **D**) are shown. **(A)** Fat-suppressed T1-weighted image demonstrating an isointense IVC LMS (black arrow) with focal areas of hypointense suggesting necrosis (white arrow). **(B)** T2-weighted image demonstrating a slightly hyperintense IVC LMS with focal areas of hyperintense suggesting necrosis (white arrow) **(C)** Diffusion-weighted image demonstrating hyperintense IVC LMS (black arrow). **(D)** Fat-suppressed T2-weighted image demonstrating a slightly hyperintense IVC LMS (white arrow) with a nodular protrusion (black arrow) within the IVC (*).

Non-contrast-enhanced MRI examinations were performed on three patients. On MRI, IVC LMS presented as a heterogeneous mass that exhibited intermediate intensity on T1-weighted images, slightly high intensity on T2-weighted images, and high intensity on diffusion-weighted images (**Figures 4A–D**). Focal areas of necrosis within the tumor appeared as low intensity on T1-weighted images and high intensity on T2-weighted images (**Figures 4A, B**). The nodular protrusion observed within the IVC on T2-weighted images was highly indicative of a tumor originating from the IVC (**Figure 4D**).

## 4 Discussion

This study revealed several CECT, US and MRI characteristics that support the diagnosis of IVC LMS. Radiologically, these tumors appeared large, lobulated, and heterogeneous on CECT, with a progressive enhancement pattern. Calcifications were rare, but necrosis was commonly observed. Additionally, most cases showed extensive formation of collaterals and varices. At the time of presentation, the IVC lumen was typically obscured on CT at the site of maximum tumor contact. On ultrasound, these tumors typically presented as lobulated, hypoechoic, and heterogeneous. On MRI, IVC LMS presented as a heterogeneous mass that exhibited intermediate intensity on T1-weighted images, slightly high intensity on T2-weighted images and high intensity on diffusion-weighted images. These findings are crucial for improving early detection and accurate pre-operative diagnosis of IVC LMS. Additionally, the observation of extensive collateral and varices formation and the typical obscuration of the IVC lumen at the tumor contact point provide important diagnostic clues that can aid in differentiating IVC LMS from other vascular abnormalities. This contributes to better clinical management and treatment planning for affected patients.

The most extensive previous research, which involved a series of 23 patients, detailed diagnostic imaging features of IVC LMS using cross-sectional imaging, although it did not include US findings (7). The previous study demonstrated that IVC LMS typically presented as a heterogeneous mass with peripheral enhancement, suggesting the presence of liquefactive necrosis (7). Our study demonstrated that the majority of IVC LMS cases showed progressive enhancement on CECT, which was associated with increased stromal fibrosis within the tumors (11). Lacomis et al. (12)suggested that fibrosis might play a significant role in the retention of contrast agents, as similar enhancement patterns have been observed in other tumors with fibrous components. Therefore, we propose that progressive enhancement might be a distinctive imaging feature of IVC LMS.

In this study, 21 patients underwent CECT, 12 underwent US and 3 underwent MRI, marking this as the largest single-institution study to evaluate IVC LMS using three kinds of imaging modalities. We observed a high incidence of necrosis (81%) in our cases, which is significantly higher than previously reported rates (38%) (13). Some tumors exhibited severe necrosis, giving them a cystic appearance. MRI is helpful in detecting necrosis, which appears as a low signal on T1-weighted images and a high signal on T2-weighted images. The majority of patients (95%) experienced local invasion into adjacent organs or vessels, and a significant proportion (76%) showed extensive collateral formation, aligning with earlier studies (7, 9). Similarly, calcification was very unusual and prior study reported that none of the tumors were calcified (7). In our study, only 2 patients (9.5%) displayed punctate calcification. Consistent with prior findings (14), the IVC lumen was typically obscured on CT at the point of maximum tumor contact. Interestingly, reports have indicated that primary IVC LMS most frequently occurs in individuals in their fifties and sixties, predominantly affecting females (15). In our study, 76% of patients were female, and the mean age was 55 years, corroborating with the literature (3, 16).

The diagnosis of intraluminal IVC LMS is typically straightforward with few differential diagnoses. On CECT, it usually appears as a large, irregular, and lobulated mass with heterogeneous enhancement within the IVC. In contrast, extraluminal IVC LMS may invade nearby organs or vessels, necessitating differentiation from other retroperitoneal tumors. However, the differential diagnoses include primary lymphoma, liposarcoma, leiomyomatosis, and gastrointestinal stromal tumors (GISTs). Primary lymphoma of the IVC typically exhibits homogeneous enhancement, distinguishing it from the heterogeneous enhancement seen in IVC LMS (17). Retroperitoneal liposarcomas, while also presenting as large heterogeneous masses, uniquely contain areas of both soft tissue and fat attenuation (18), features not seen in IVC LMS. Intravenous leiomyomatosis generally extends unilaterally through the common iliac veins into the IVC and may involve intracardiac extension with associated uterine leiomyomas (19). Unlike IVC LMS, GISTs typically appear in submucosal or intraluminal positions within the gastrointestinal tract and often exhibit mutations in the KIT tyrosine kinase receptor gene, express the KIT (CD117) protein, and respond to tyrosine kinase inhibitors (20). Overall, accurate interpretation of these imaging findings can enhance clinical decision-making, influences treatment outcomes, and ultimately can improve patient survival rates by ensuring that each condition is managed with the most appropriate and effective interventions.

Our study is subject to several limitations. Firstly, despite having a larger cohort than previous studies, the sample size remains relatively small, which may limit the generalizability of our findings. Secondly, the retrospective nature of the study could introduce biases related to the selection of cases and the availability of data. Thirdly, only half of the patients underwent ultrasonography and three underwent MRI, which means our understanding of the ultrasound and MRI characteristics of IVC LMS is incomplete. This limitation potentially restricts a comprehensive comparison between imaging modalities and might affect the reliability of US as a diagnostic tool in this context. Fourthly, no patient underwent PET-CT scan before surgery. PET-CT can be used for assessing distant metastases and tumor TNM staging and to differentiate IVC LMS from inferior vena cava thrombosis. Fifthly, our study lacks data from contrast-enhanced MRI and contrast-enhanced ultrasonography, which could have been used to differentiate between an intraluminal mass and a thrombus, as well as to assess the degree of obstruction and the extent of collateral circulation development. Finally, our study did not include a comparative analysis of the CECT, US and MRI findings of IVC LMS with those of other similar tumors. Such a comparison could have provided additional insights into the distinct or overlapping imaging features of IVC LMS relative to other neoplastic conditions, thereby aiding in differential diagnosis.

## 5 Conclusion

In summary, our study has identified distinct imaging characteristics of IVC LMS on CECT and US, which can be delineated as follows: (a) the appearance of a large, lobulated, and heterogeneous mass that often exhibits necrosis on CT scans; (b) a pattern of progressive enhancement observed on CECT, indicative of increased stromal fibrosis of these tumors; (c) the presence of extensive collateral formation and varices, highlighting the impact of the tumor on the surrounding vascular structures; (d) a lobulated, hypoechoic mass with IVC obstruction on US. These imaging signatures are crucial for radiologists and sonographers in diagnosing IVC LMS accurately. Given the aggressive nature of these tumors and their tendency to worsen rapidly, their prognosis remains poor. Hence, achieving early diagnosis is critical, and it significantly hinges on the meticulous analysis of imaging results. This underscores the importance of early and precise imaging diagnosis in managing patients with IVC LMS effectively.

## Data Availability

The original contributions presented in the study are included in the article. Further inquiries can be directed to the corresponding author.
